# Arabo-Farsi anatomy figures

**DOI:** 10.4103/0256-4947.51809

**Published:** 2009

**Authors:** Farid Sami Haddad

**Affiliations:** Custodian of the Sami I Haddad Memorial Library, Rancho Palos Verdes, California, USA haddadmd@cox.net

*We conclude our series of articles on the “Anatomy Charts”^1^–^4^ with the following note from Dr. Haddad. The images and accompanying description may now be found on Wikipedia:* (http://en.wikipedia.org/wiki/Anatomy_Charts_of_the_Arabs)

**To the Editor:** The table and references below summarize available information on 17 sets of very similar anatomy figures that I have been able to glean from the current published literature and put at the disposal of the readers. It is fair to assume that there are a few more similar figures, the locations of which are not yet known. I urge the reader and expressly enlist his kind cooperation in the task of completing this collection, which, at present, stands at exactly 100 colored hand-painted anatomy figures.

There are several disputed points and questions about these figures. Here are some of them:

1. Why did a few historians assume that these figures might be of Scythian or Tibetan origin?
2. Which set is the original and which are copies?
3. Who is the author of these figures?
4. Why is the head always represented in a Mongolian perspective and why was the squatting position chosen?
5. Why do these figures usually (with the exception of three sets) appear in mas ‘wd's text on anatomy?
6. Why aren't these figures always mentioned or referred to in the text in which they are found?
7. Why do they, sometimes, seem to have been just bound within the volume containing them and do not seem to form an integral part of it?
8. Are they all from the same hand or drawn by different scribes?
9. What is their relation to the similar Latin figures?

**Table 1:** Seventeen sets of anatomy figures

No	Date	Author	Place	Number	References
01	<1400 AD	manSwr	Paris	1555	5, 13, 14
02	<1400 AD	manSwr	London	23556	5
03	1400 AD	manSwr	Oxford	1576	5, 6
04	C 1400 AD		India Office	2296	5, 6
05	C 1450 AD	manSwr	Bethesda		9, 10
06	C 1450 AD	manSwr	Bethesda		9, 10
07		manSwr	New Haven		10
08		manSwr	New Haven		10
09			New Haven		10
10		Ibn sÿnA	London		17
11		Ibn sÿnA	London	
12	1537 AD	mahAbAdÿ	California	90	8, 11, 15, 16
13	C 1650 AD	manSwr	Durham		12, 18
14	C 1650 AD	manSwr	Kansas		10
15	C 1650 AD	manSwr	Montreal	7785, 75	7, 10
16	C 1750 AD	manSwr	Kansas		10
17	C 1850 AD	manSwr	Montreal	7785, 76	7, 10

All manuscripts have six figures except number 12, which has only four.

Once we have the answers to these questions, one can hope to study the available data with a comparative and critical analysis so that some of the existing mysteries can be resolved.
